# When music “flows”. State and trait in musical performance, composition and listening: a systematic review

**DOI:** 10.3389/fpsyg.2015.00906

**Published:** 2015-06-30

**Authors:** Alice Chirico, Silvia Serino, Pietro Cipresso, Andrea Gaggioli, Giuseppe Riva

**Affiliations:** ^1^Applied Technology for Neuro-Psychology Lab, IRCCS Istituto Auxologico Italiano, Milan, Italy; ^2^Department of Psychology, Università Cattolica del Sacro Cuore, Milan, Italy

**Keywords:** flow experience, music, state flow, trait flow, dispositional flow, systematic review, PRISMA

## Abstract

It is not unusual to experience a sense of total absorption, concentration, action-awareness, distortion of time and intrinsic enjoyment during an activity that involves music. Indeed, it is noted that there is a special relationship between these two aspects (i.e., music and flow experience). In order to deeply explore flow in the musical domain, it is crucial to consider the complexity of the flow experience—both as a “state” and as a “trait.” Secondly, since music is a multifaceted domain, it is necessary to concentrate on specific music settings, such as (i) musical composition; (ii) listening; and (iii) musical performance. To address these issues, the current review aims to outline flow experience as a “trait” and as a “state” in the three above-mentioned musical domains. Clear and useful guidelines to distinguish between flow as a “state” and as a “trait” are provided by literature concerning flow assessment. For this purpose, three aspects of the selected studies are discussed and analyzed: (i) the characteristics of the flow assessments used; (ii) the experimental design; (iii) the results; and (iv) the interrelations between the three domains. Results showed that the dispositional approach is predominant in the above-mentioned settings, mainly regarding music performance. Several aspects concerning musical contexts still need to be deeply analyzed. Future challenges could include the role of a group level of analysis, overcoming a frequency approach toward dispositional flow, and integrating both state and dispositional flow perspectives in order to deepen comprehension of how flow takes place in musical contexts. Finally, to explain the complex relationship between these two phenomena, we suggest that music and flow could be seen as an emergent embodied system.

## Introduction

Take Il Volo, the popular trio of Italian pop-opera singers, who won the top prize at the Sanremo Music Festival in 2015. They begin to sing, and each of them seems to know exactly what to do and when to do it. Everything flows easily even though the piece of music is clearly difficult to perform.

Now think of a time when you were involved in singing or playing an instrument, or simply in listening to music. You will probably remember that time seemed to stop or to accelerate; you were totally concentrated on the music; everything flowed easily and you felt a sense of joy and fulfillment.

Even though not all the above-mentioned conditions are always experienced by all people in every circumstance, when some of them occur, we experience a feeling similar to happiness.

Within the paradigm of Positive Psychology (for a review, see Ryan and Deci, 2001), which emphasizes the role of well-being in human life, the above-mentioned feelings are considered crucial elements to promote well-being both in terms of satisfaction regarding life, presence of positive moods (i.e., subjective well-being; Diener et al., 1999) and self- actualization (i.e., psychological well-being; Ryan and Deci, 2000).

Csikszentmihalyi (1975), in the course of interviewing people engaged in pleasurable and intrinsically-motivating activities and trying to understand the resulting unique experience, discovered the existence of a multidimensional phenomenon called “flow.” He described flow as “the holistic sensation that people feel when they act with total involvement” (Csikszentmihalyi, 1975, p. 36) and noted that it was characterized by a strong correlation with well-being (Delle Fave et al., 2011).

To explain further, flow experience (Csikszentmihalyi, 1975, 1990) is a state of full engagement, control, concentration and action awareness, occurring during an activity perceived as highly self-rewarding and characterized by clear goals, unambiguous feedback, distortion of time perception, loss of self-consciousness and a balance between challenges and skills required to best perform it. These characteristics of flow are also the nine dimensions this experience is composed of (Csikszentmihalyi, 1975).

Moreover, Csikszentmihalyi (1990, 1993) found out that artists and athletes seemed more likely to experience flow, especially during their work. In light of this premise, this concept has been studied mainly in three settings: sports (Muzio et al., 2012; Swann et al., 2012), work (Csikszentmihalyi and Csikszentmihalyi, 1992; Nakamura and Csikszentmihalyi, 2009), and music (O’Neill, 1999; MacDonald et al., 2006; Diaz, 2013; Fullagar et al., 2013; Wrigley and Emmerson, 2013; Hart and Di Blasi, 2015).

The musical domain is particularly interesting because it is indisputable that music is very important to humans. Even as fetuses, people have the ability to hear and respond to music (Lecanuet and Schaal, 1996; Trevarthen, 2002; O’Neill, 2005). The relevance of music has also been noted especially in adulthood, mainly in the third age (Gembris, 2008). It causes people “to become a little bit more real” (Gembris, 2008, p. 107), helping to improve quality of life, happiness, health and sense of community. We are never without music, even if we are not concentrated on it in a specific moment (Frith, 2002). Finally, it has a great impact on our lives, mainly in promoting our well-being (Thorgaard et al., 2004; Hays and Minichiello, 2005).

According to the framework of Positive Psychology (Seligman and Csikszentmihalyi, 2000), Csikszentmihalyi himself introduced the idea that music and flow are strictly linked, mainly because music can sustain people’s intrinsic motivation, which is one of the main features of flow experience (Csikszentmihalyi, 1975, 2000, 1997). He theorized and investigated this relationship, considering music among other “leisure activities” (i.e., singing, playing an instrument alone or in a group), and he posited that music is an activity in which it is easier to reach an experience of flow (Csikszentmihalyi, 1997). Indeed, music is an activity that fosters flow more often than other activities (Lowis, 2002). Therefore, his analysis of the relationship between these two pillars of well-being began as an investigation into how flow is displayed and emerges in musical activities.

The analysis of flow in musical contexts is a rapidly-developing field as suggested in Croom (2012, 2015) due to the complexity of both phenomena (i.e., flow and music), which has led scholars to focus on several different aspects of them, such as emotions (Lamont, 2012; Marin and Bhattacharya, 2013); motivation (Csikszentmihalyi and Rich, 1997; Schmidt, 2005; Karageorghis et al., 2008; Digelidis et al., 2014); performance anxiety management (Wilson and Roland, 2002; Kirchner, 2011; Fullagar et al., 2013); social relationships (Custodero, 2002; Bakker, 2005; Bloom and Skutnick-Henley, 2005; Freer and Raines, 2005; Freer, 2009; Hart and Di Blasi, 2015); creativity (Csikszentmihalyi, 1997; Sheridan and Byrne, 2002; MacDonald et al., 2006) and psychophysiological correlates of flow experience (de Manzano et al., 2010). Further, according to the aim of each study, flow was studied in relation to different populations of musicians and non-musicians involved in musical activities (Custodero, 2002, 2005, 2012; Bailey and Davidson, 2005; Bakker, 2005; Biasutti and Frezza, 2009; Freer, 2009; de Manzano et al., 2010; Nijs et al., 2012; Wrigley and Emmerson, 2013).

On the other hand, the majority of the studies regarding music and flow refer to music in terms of “musical activities,” as Csikszentmihalyi himself first suggested (Csikszentmihalyi, 1997). Indeed, according to literature, the most investigated musical activities in relation with flow are (i) musical performance (O’Neill, 1999; Sawyer, 2006; Kirchner, 2011); (ii) musical composition (Byrne et al., 2003; John, 2006; MacDonald et al., 2006; Hart and Di Blasi, 2015); and (iii) listening (Lamont, 2012; Diaz, 2013), as Marin and Bhattacharya clearly evidenced (Marin and Bhattacharya, 2013). With regard to listening, researchers were able to expand the field, exploring the effects of music also in non-musical contexts, such as sports (Pates et al., 2003; Laukka and Quick, 2013) and online environments (Grice and Hughes, 2009).

However, to date, despite the growing body of research regarding this theme, and maybe because of the heterogeneity that characterizes this field, there has been only a weak attempt to organize the findings reached up to now. Even so, there are several anchors that have clearly emerged from all the literature concerning flow and music. For example, it is noted that flow and performance anxiety are two antithetical phenomena (Kirchner, 2011; Fullagar et al., 2013). Another fundamental point is that flow in music can be considered as a “motivator” especially for young musicians (Csikszentmihalyi and LeFevre, 1989; O’Neill, 1999) and that it has a strict relationship with positive emotions according to a Eudaimonic perspective (Seligman, 2002; Juslin and Sloboda, 2010; Lamont, 2012).

Finally, it is also noted that flow itself can be analyzed in musical activities from at least two different perspectives. More specifically, flow experience can be considered both a “state” (something related to circumstances; Csikszentmihalyi, 1975, 2000) and a “trait” (something depending on one’s personality; Csikszentmihalyi, 1993). In other words, flow is not only a transient experience but also a predisposition that depends on individual differences (Keller and Blomann, 2008; Mosing et al., 2012). Csikszentmihalyi himself suggested this last possibility, introducing the concept of “autotelic personalities: the mark of the autotelic personality is the ability to manage a rewarding balance between the “play” of challenge-finding and the “work” of skill-building (Csikszentmihalyi, 1993, p. 80). Some people (those with autotelic personalities) might be more prone to flow experiences than others. Autotelic personalities are composed of opposite personality traits, such as curiosity and persistence; the ability to concentrate deeply but also to be open to novelty; and independence in conjunction with cooperation (Nakamura and Csikszentmihalyi, 2009). These personality characteristics can help people handle the complex interplay between challenges and skills. Therefore, flow can’t be considered only an experience related to immediate specific situations, namely a “state.” It is also a peculiar characteristic of some people (i) who manage the balance between challenges and skills more successfully than others; (ii) who are prone to search for new challenges; and (iii) who are actively engaged in facing such challenges (Csikszentmihalyi, 1993).

In sum, an autotelic personality can be thought of as the union of both “receptive (e.g., openness) and acetive qualities (e.g., engagement and persistence)” (Baumann, 2012, p. 3).

At a methodological level, the emerging questions regarding autotelic personalities are (i) how to measure the individual proneness to experience flow and (ii) which internal and relatively stable individual characteristics are related to the merging of flow (Keller and Blomann, 2008; Baumann, 2012; Mosing et al., 2012; Marin and Bhattacharya, 2013).

To address these issues, researchers adopted two methodological approaches. The first refers to the assessment of flow frequency and intensity in an individual’s life (Csikszentmihalyi, 1975, 1993, 1997, 2000; Jackson and Eklund, 2004; Jackson et al., 2008); the second aims to infer personality traits that characterize high-performance people compared with average individuals (Jackson, 1974; Csikszentmihalyi, 1997).

While the second approach is totally absent in the musical domain, the first is often employed in musical contexts in which occurrence and intensity of flow experience in individuals’ lives have been recently studied in relation with other personality traits (Fritz and Avsec, 2007; Jackson et al., 2008; Asakawa, 2010; Ullén et al., 2012; Marin and Bhattacharya, 2013; Butkovic et al., 2015).

Our review did not concentrate only on stable causes of flow but also aimed to consider flow as a “state,” namely something related to specific types of activities categorized as high or low flow-conductive (e.g., Bakker, 2005; Diaz and Silveira, 2013).

Flow as a “state” focuses mainly on specific contexts, activities or external contextual characteristics and elements that are able to foster this optimal experience; there is less emphasis on internal factors like personality traits (Bakker, 2005; Bryan-Kinns and Hamilton, 2012).

According to these premises, it is possible to distinguish two approaches of study toward flow: flow as a “state” and flow as a “trait.”

Clear and useful guidelines to distinguish between flow as a “trait” and as a “state” are provided by literature concerning flow assessment.

Indeed, according to Jackson and Eklund (2002), Jackson et al. (2008, 2010), Martin and Jackson (2008), and Jackson and Marsh (1996), it is possible to identify four main instruments to assess flow as a state (i.e., Flow State Scale; Flow State Scale-2; Short; and Core State Flow Scales) and four to assess flow as a trait (i.e., Dispositional Flow Scale; Dispositional Flow Scale-2; Short; and Core Dispositional Flow Scales; Swedish Flow Proneness Questionnaire).

Finally, these two aspects of flow reflect its complex and multifaceted nature, which emerges in several domains, such as sports (Muzio et al., 2012; Swann et al., 2012), work (e.g., Csikszentmihalyi and LeFevre, 1989; Eisenberger et al., 2005; Nakamura and Csikszentmihalyi, 2009), and music (O’Neill, 1999; MacDonald et al., 2006; Lamont, 2012; Diaz, 2013; Fullagar et al., 2013; Wrigley and Emmerson, 2013; Hart and Di Blasi, 2015). However, the analysis of flow in music performance, listening and composition has received little attention compared to the other two domains (Marin and Bhattacharya, 2013).

Therefore, as each of the two above-mentioned approaches (trait and state) gives a unique contribution to a better understating of flow, the aim of this review is to present the implications of adopting one, the other or both perspectives to investigate flow in three above-mentioned most-investigated music domains, as suggested also by (Marin and Bhattacharya, 2013), namely: (i) composition, (ii) listening and (iii) music performance.

In particular, in the current review we aim to organize findings regarding a small but significant proportion of studies concerning flow and music. We focused on flow as a trait and as a state in musical contexts in order to provide initial but solid guidelines for future research in this field.

## Materials and Methods

We followed the Preferred Reporting Items for Systematic Reviews and Meta-Analysis (PRISMA) guidelines (Moher et al., 2009).

### Search Strategy

To achieve this, a computer-based search in several databases was performed for relevant publications. Databases used for the search were PsycINFO, Web of Science (Web of Knowledge) and PubMed.

The search string was: (“music”) AND (“flow” OR “flow experience” OR “trait flow” OR “state flow” OR “dispositional flow.”

The articles were individually scanned to elaborate whether they fulfilled the following inclusion criteria: (i) research article; (ii) providing information about the used sample; and (iii) providing information about measures. These inclusion criteria were used for several reasons. As noted above, information about the sample and measures are a prerequisite.

#### Inclusion Criteria

With this purpose, we chose two inclusion criteria in this review. First, studies were selected based on the fact that they used instruments of flow assessment. Therefore, studies that considered flow as an object of analysis were included.

In order to distinguish studies that analyzed flow as a trait, as a state or as both of them, without ambiguity, we referred to literature concerning flow assessment (Jackson and Marsh, 1996; Jackson and Eklund, 2002; Jackson et al., 2008, 2010; Martin and Jackson, 2008), which identified four main instruments to assess flow as a state (i.e., Flow State Scale; Flow State Scale-2; Short; and Core State Flow Scales) and four to assess flow as a trait (i.e., Dispositional Flow Scale; Dispositional Flow Scale-2; Short; and Core Dispositional Flow Scales).

Secondly, starting from evidences of literature (O’Neill, 1999; Byrne et al., 2003; Pates et al., 2003; John, 2006; MacDonald et al., 2006; Sawyer, 2008; Grice and Hughes, 2009; Kirchner, 2011; Lamont, 2012; Diaz, 2013; Diaz and Silveira, 2013; Laukka and Quick, 2013; Hart and Di Blasi, 2015) and from suggestions provided by (Marin and Bhattacharya, 2013) we included only researches which considered three music domains:

(i) Composition: target was involved in a music composition task;
(ii) Listening: target was asked to listen to one or more excerpts of music;
(iii) Music performance: target concerned conductors, people who played an instrument and sang (amateurs or experts).

Because of the heterogeneity that characterizes this field, we decided not to select studies on the basis of the target. Therefore, we included both musicians and non-musicians.

We decided to focus on flow because of the solid theoretical and methodological background supporting this experience (Jackson and Marsh, 1996; Jackson and Eklund, 2002; Jackson et al., 2008, 2010; Martin and Jackson, 2008). Further, we found evidence that flow, peak experiences and peak performances are different phenomena even though they share some aspects (Privette, 1983; Privette and Bundrick, 1991). Because the aim of this review is not clinical, we excluded music therapy studies.

**FIGURE 1:**
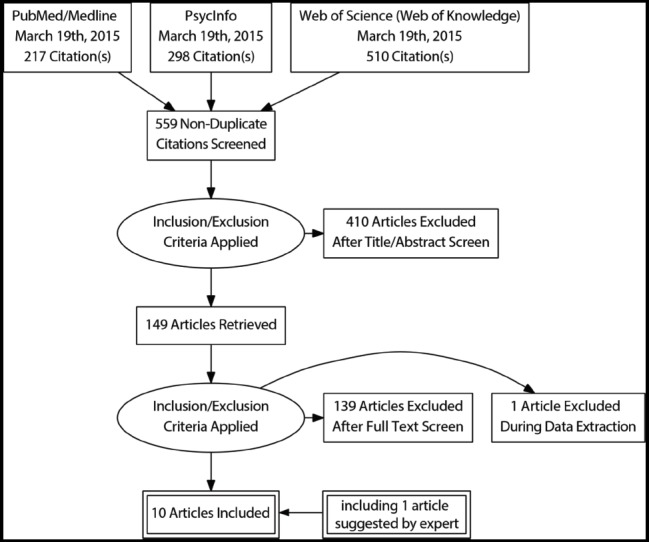
**Search strategy flowchart**.

After the application of the inclusion criteria, papers were reduced to 149. A deeper investigation of the full papers resulted in the exclusion of 139 more articles. During the data extraction procedure, 1 additional full paper was excluded. In the end, 9 studies met the full criteria. Further, more Expert researchers in the field were contacted for suggestions on further studies considered in our research. One new study was suggested and has been included in the analyzed studies. Finally, 10 studies were included in this review (see Table 1). A flowchart showing the procedure is detailed in Figure 1.

**TABLE 1:** **Information about the selected studies on flow and music**.

**Study**	**Sample**	**Experimental design**	**Condition(s)**	**Session(s)**	**Instruments of Flow assessment**	**Flow perspective**	**Outcomes**
Wrigley and Emmerson (2013)	236 students attending music performance programs at a conservatorium of music of an Australian university	Confirmatory Factor Analysis for the validation of an instrument of Flow assessment	1 condition: Flow State Scale-2	1 session	Flow State Scale-2 (Jackson and Eklund, 2002; Jackson et al., 2008)	State flow	Low levels of flow among participants after examinations and no correlation between flow and several characteristics of context was found.
Pates et al. (2003)	3 female netball players	A single- subject multiple baselines across individuals design	11 conditions	11 sessions (trials) of shooting and assessment	Flow State Scale (Jackson and Marsh, 1996; Jackson and Eklund, 2004)	State flow	All athletes improved their performance across 11 trials. Flow levels of only two athletes in three increased.
Nijs et al. (2012)	65 musicians (professional and amateur)	Correlational	No conditions	3 sessions (assessment- interaction with Music Paint machine- assessment)	Flow State Scale (Jackson and Marsh, 1996; Jackson and Eklund, 2004)	State flow	Music Paint machine is flow conductive
Sinnamon et al. (2012)	125 students of music (amateur and elite) attending two conservatories	Between subject design	2 conditions	1 session	Dispositional Flow State Scale-2 (Jackson and Eklund, 2002; Jackson et al., 2008)	Dispositional or trait flow	DFS-2 showed a high reliability and validity in this musical context
Fritz and Avsec (2007)	84 student of music at the Music Academy of Ljubljana	Correlational	No conditions	1 sessions	Dispositional Flow Scale-2 (Jackson and Eklund, 2002, 2004) translated in Slovene	Dispositional or trait flow	Flow experience showed low correlation with satisfaction with life, except for dimensions of action-awareness, challenge and skills balance and autotelic experience. No correlation between any dimension of well being and “Loss of self-consciousness” and “time transformation” was found. Flow correlated especially with emotive dimensions of well being.
Marin and Bhattacharya (2013)	76 piano performance students	Correlational study with prediction of trait emotional intelligence and amount of daily practice on flow	2 conditions (high vs. low performers)	1 session	Dispositional Flow Scale-2 (Jackson and Eklund, 2002, 2004)	Dispositional or trait flow	Amount of daily piano practice and level of trait emotional intelligence predicted flow. In details, flow did not correlate with high achievements (as winning a prize in piano competition) and emotive aspects of music seemed to be able to support flow merging.
de Manzano et al. (2010)	21 professional pianists	Within subject design	5 trials	Warm up period-musical performance and	Short Flow State Scale (Jackson and Eklund, 2004)	State flow	Flow seemed to be related to a parasympathetic activity which can influence
Baker and MacDonald (2013)	30 participants (15 retireers and 15 university students)	Within subject design	3 conditions [lyric creation (LC); song parody (SP); and original songwriting (OS)]	Each condition was scheduled on separate days and was always followed by a Flow assessment.	Short Flow State scale and Core Dispositional Flow Scale (Martin and Jackson, 2008)	State and dispositional or trait flow	No significant difference among conditions and between students and retireers concerning both flow as a trait and a as a state was found. At a global level, state and trait flow correlated with questionnaire scores concerning song creation and Core Dispositional Flow Scale showed the highest correlation with the questionnaire scores. Dispositional flow influenced positively the process of song creation.
Martin and Jackson (2008)	22 4 young classical musicians attending high school	Study for the validation of Flow brief measure	No conditions	1 session	First study: Short Flow State Scale in a musical context	Dispositional or trait Flow	From CFA emerged an acceptable fit (Chi- square = 45.11; df = 27).
Butkovic et al. (2015)	10,699 twins aged between 27 and 54.	Correlational study with prediction of intelligence, personality, motivation and flow proneness on music practice	No conditions	1 session	Swedish Flow Proneness Questionnaire (SFPQ)	Dispositional or trait flow	Music flow predicted music practice and it depended more on genetic than situational factors.

To assess the risk of bias, PRISMA recommendations for systematic literature analysis have been strictly followed. Three authors (AC, SS, and PC) independently selected paper abstracts and titles and analyzed the full papers that met the inclusion criteria, resolving disagreements through consensus.

## Results

Despite the growing body of studies investigating flow in sports (Muzio et al., 2012; Swann et al., 2012) and work (e.g., Csikszentmihalyi and LeFevre, 1989; Nakamura and Csikszentmihalyi, 2009; Engeser, 2012), to date few researches have been concentrated on the musical domain, even though music was demonstrated to elicit flow most often (Lowis, 2002). However, a lot of progress has been made in this specific field.

Therefore, the current review aims to organize findings regarding flow as a trait and as a state in musical contexts in order to provide initial but solid guidelines for future research in this field. To this purpose, we analyzed: (i) the characteristics of the flow assessments used; (ii) the experimental design; (iii) the results; and (iv) interrelations across the three domains. We were interested mainly in findings reached by each study concerning both of the above-mentioned perspectives of flow (i.e., flow as a “trait” and as a “state”) in the three musical domains of musical composition, listening and musical performance, in order to provide an initial but solid background for future research in this field.

For each of the three above-mentioned domains of music (i.e., musical composition, listening and music performance), studies concerning flow as a “trait” or as a “state” are presented. Distinction between the two approaches toward flow was made regarding literature concerning flow assessment instruments (Jackson and Marsh, 1996; Jackson and Eklund, 2002; Jackson et al., 2008, 2010; Martin and Jackson, 2008).

### Flow Assessment Instruments as a “trait” and as a “state”

According to (Jackson and Marsh, 1996; Jackson and Eklund, 2002; Jackson et al., 2008, 2010; Martin and Jackson, 2008) it is possible to assess flow from two different approaches: (i) considering Flow as a state or (ii) as a disposition (i.e., a trait).

Clear and useful guidelines to distinguish between flow as a “trait” and as a “state” are provided by these studies, which concerned eight main flow assessment instruments.

(i) Flow State Scale (FSS; Jackson and Marsh, 1996) and Flow State Scale-2 (FSS-2; Jackson and Eklund, 2002; Jackson et al., 2008, 2010).
(ii) Dispositional Flow Scale (DFS; Jackson et al., 1998) and Dispositional Flow Scale-2 (DFS-2; Jackson and Eklund, 2002; Jackson et al., 2008, 2010).
(iii) Core Flow State Scale (C FSS) and Core Dispositional Flow Scale (C DFS; Jackson et al., 2008, 2010; Martin and Jackson, 2008).
(iv) Short Flow State Scale (S FFS) and Short Dispositional Flow Scale (S DFS; Jackson et al., 2008, 2010; Martin and Jackson, 2008).

Two long and multidimensional scales to assess flow in terms of a “state” (Jackson and Marsh, 1996) are present in literature. They are both self-reported 36-item scales on 5-point answer scale, in which each item refers to one of the nine components of flow (Csikszentmihalyi, 1990; Jackson and Csikszentmihalyi, 1999). They showed good reliability and represented a retrospective measure of flow concerning a specific experience, measuring the intensity of the experience of each dimension of flow.

There also exist two long and multidimensional scales to assess flow in dispositional terms, namely, the Dispositional Flow Scale (Jackson et al., 1998) and the Dispositional Flow Scale-2 (Jackson and Eklund, 2002). Both scales measure the individual proneness to experience of flow. In particular, they assess the frequency of flow experience dimensions in the life of an individual.

There are also two “one-dimensional” measures of flow and two “Core” measures of flow. They were developed from the two above-mentioned multidimensional scales (Flow State Scale and Dispositional Flow Scale). While the Short Flow State Scale and Short Dispositional Flow Scale are, effectively, a short version of the two above-mentioned scales (Jackson and Eklund, 2002; Jackson et al., 2008; Martin and Jackson, 2008) the Core Flow State Scale and Core Dispositional Flow Scale aim to capture the central features of flow experience (Martin and Jackson, 2008), overcoming the above-mentioned dimensional flow-model (Jackson et al., 2010).

The aforementioned instruments were used as guidelines to select and classify studies according to the perspectives of flow as a “state” and as a “trait.”

### Musical Composition

Musical composition mainly concerns the creation of new excerpts of music starting from improvisation or from a longer process of music creation. (Baker and MacDonald, 2013) provided an example of the second case in a therapeutic context. They explored the songwriting process in terms of comfort in personal sharing, sense of self, personal and collective identity, song ownership, achievement and song satisfaction.

Finally, they concentrated on implications of songwriting regarding flow experience. Following a within subject design, they randomly assigned participants (15 university students and 15 retirees) to three conditions: (i) creating lyrics; (ii) writing a song parody; and (iii) original songwriting. After participants had been engaged in the process of songwriting, their level of flow as a trait (i.e., Core Dispositional Flow Scale) and a state (i.e., Short Flow State Scale; Jackson et al., 2008; Martin and Jackson, 2008) and their feelings toward songwriting were assessed. Results showed that students and retirees experienced a similar level of flow. On the other hand, retirees found the songwriting process less full of significance than the students did, even though flow disposition of both students and retirees predicted their attitude toward the songwriting process as a whole (*R*^2^= 0.20). Therefore, age did not emerge as a factor determining the likelihood of experiencing flow. Nevertheless, it could influence how meaningful the original songwriting process is perceived as. Finally, the process of creation (of parody, of lyrics or of original songs) appeared to be more flow-conductive (*r* = 4.18–4.41) than performing each musical product (*r* = 3.47), according to literature (Jackson and Eklund, 2004).

An example of music composition through improvisation is provided by (Nijs et al., 2012) Researchers analyzed the interaction of 65 musicians (professional and amateur) with a Music Paint machine (i.e., an interacting music system “allowing a musician to make a painting on a computer screen by playing an acoustic music instrument and by moving on a colored pressure sensing mat;” (Nijs et al., 2012, p. 2) in terms of flow and presence (Biocca and Harms, 2003; Riva, 2008; Riva et al., 2011). This innovative and interactive music system allowed feedback to be received during the whole process of musical composition.

Results showed that this innovative music system was flow-conductive and that the emergence of an experience of flow was mainly due to the occurrence of high levels of sense of presence, which is the sense of being able to realize our own intentions in a real or virtual world (Biocca and Harms, 2003; Riva, 2008; Riva et al., 2011). In other words, flow emerged as an embodied phenomenon that is strictly related to body for its occurrence, as we discuss in the conclusion. Clear feedback and a deep sense of control in conjunction with a sense of action awareness are the keys to tune our actions with environmental perception.

#### Listening

Despite the growing interest surrounding flow and music listening (Lamont, 2012), we found only one study concerned with the effect of listening to music on flow levels of participants, according to our guidelines. Indeed, this study used a flow state assessment instrument according to guidelines provided by (Jackson and Marsh, 1996; Jackson and Eklund, 2002; Jackson et al., 2008, 2010; Martin and Jackson, 2008).

Pates et al. (2003) used the extended version of FSS (Jackson and Marsh, 1996) to test flow variations. They considered the effect of music selected by three netball players on their level of flow and performance. Previously, they explained flow theory to participants and asked them to select an excerpt of music related to flow experience (i.e., “self-selected asynchronous music,” Pates et al., 2003, Abstract). This meant that participants listened to music selected by themselves before each performance, not during its execution. Players listened to this self-selected excerpt of music each time they were attempting a shot (11 trials in all). Researchers used the FSS to assess flow after each of the 11 trials, showing that improvements of performance were associated with music but not with flow levels. In fact, during the 11 trials, each of the three netball players showed an increase in performance, but only two players showed an increase in flow levels. According to this study, flow levels increased across the 11 trials, but only for two of the three netball players. Therefore, high performance levels were not necessarily associated with flow experience, but seemed to be supported by self-selected asynchronous music. Maybe the relationship between flow and performance did not emerge clearly because of the small samplesize. For this reason, it could be necessary to also assess individual differences concerning flow proneness in order to have a more organic perspective of analysis.

#### Musical Performance

The majority of studies included in this review concentrated on the domain of musical performance. de Manzano et al. (2010) focused on psychophysiological correlates of flow during music performance. In this research, a “state” approach of flow assessment was adopted. de Manzano et al. (2010) employed a brief measure of flow, the “Short Flow Scale” (Jackson and Eklund, 2004), to assess psychophysiology correlates of flow in expert pianists after their performance. The peculiarity of this study was the fact that flow participants’ level was measured five times in order to assess flow fluctuations and to take into account the assumption that this experience could be long-lasting and varied in time. The context was formal though not “competitive.” In this study it was demonstrated that flow, at a global level, did not show significant fluctuations during a repetitive activity, such as playing the same excerpt of music five times. On the other hand, they found that the attention paid to each performance (i.e., dimension of “concentration”) and the intrinsic motivation felt during music execution (i.e., dimension of “autotelic experience”) showed significant variations across trials. Therefore, interplay between the emotive and cognitive aspects of flow could be interpreted as the key determinants of this experience. Finally, regarding physiological parameters, it seemed that during performance execution, flow was associated with a parasympathetic activity that modulated sympathetic activity (de Manzano et al., 2010, p. 307).

The interest for state and trait components of flow in musical contexts seems to be growing as of late. In fact, it is possible to find several studies that aimed to validate dispositional and state flow instruments in musical contexts (Martin and Jackson, 2008; Sinnamon et al., 2012; Wrigley and Emmerson, 2013).

Wrigley and Emmerson (2013) extended the target considered by de Manzano et al. (2010)by including different types of musicians (i.e., string and piano performers, woodwind, voice, and brass performers) to empirically validate FSS-2 (Jackson and Eklund, 2002, 2004; Jackson et al., 2008). Therefore, this research also concerned a state perspective of flow. In particular, this study took place in a competitive environment and in a specific musical setting (i.e., two semesters of examinations at a conservatory of music at an Australian university). Variables concerning gender, semester or year level and type of instrument played did not emerge as influencing flow perception. Only piano players showed significantly low flow scores compared with other types of instruments. Results evidenced (i) the high reliability of FSS-2 in measuring flow also in live musical contexts (Cronbach’s alpha raged between 0.81 and 0.92); and (ii) the goodness of the nine-dimensional model measured using FSS-2 (Chi-square: 754.53; *p* < 0.01). In other words, flow experience emerged as a complex and multifaceted phenomenon also in the context of musical performance. Therefore, not only state factors of flow but also individual flow proneness played a key role in this kind of situation. Indeed, this study suggested that in examination settings, flow seemed to be related to an individual disposition instead of other conditions.

Therefore, this research evidenced the possibility that not only factors related to context, such as year and type of instrument played, can influence flow, but also dispositional ones.

Martin and Jackson had explored this possibility, validating a brief measure of dispositional flow. While the Core Flow State Scale was never used to study the relationship between flow and music, Jackson et al. (2008) tested the Short Flow Scale (9-item scale on a 7-point answer scale) specifically in a live musical context and in relation to motivation and engagement. They considered a heterogeneous target [violin (20% of respondents), piano (19%), clarinet (9%), flute (8%), cello (6%), voice (6%), trumpet (5%), and 5% other instruments]. They found a confirmation for the nine-item model in musical contexts also from a dispositional perspective (Chi-square: 45.11; df = 27).

The same results were reached by Sinnamon et al. (2012), who validated another flow assessment instrument, the dispositional Flow Scale-2 (Jackson and Eklund, 2002, 2004; Jackson et al., 2008), in a musical context. Participants were distinguished on the basis of their musical experiences (i.e., amateurs vs. elite students). DFS-2 showed high reliability according to both amateurs and elite students (amateur students: 0.89; elite students: 0.92). The nine-dimension model showed a good fit, while some sub-dimensions were less related to global flow than others (0.46; *p* < 0.01 for “loss of self-consciousness” dimension to 0.74; *p* < 0.01 for “autotelic experience”).

Finally, amateur students experienced flow less frequently (87%) than elite students did (95%), even though this difference was found not to be significant by a *t*-test; amateur students also experienced higher “loss of self-consciousness” levels than elite students did. In general terms, the dimension of “loss of self-consciousness” was the lowest with respect to other subscales. This result is the same as the one obtained by Marin and Bhattacharya (2013), who assessed the relationship between trait emotional intelligence (TEIQue-SF; Petrides and Furnham, 2006), daily amount of practice, and flow in dispositional terms (DFS-2) in the specific context of music (using two self-reported questionnaires created ad hoc). In this study, DFS-2 showed a high reliability (*α* = 0.89). They started from the findings of Wrigley and Emmerson (2013), which indicated that pianists (mean = 3.93, SD = 0.49) had a lower flow score relative to brass (mean = 4.36, SD = 0.55) and string players (mean = 4.22, SD = 0.56). Therefore, they concentrated on pianists and discovered that their daily amount of practice and trait emotional intelligence predicted flow (adjusted R^2^ = 0.27). Further, amount of practice is closely related to high performance levels, while, according to (Pates et al., 2003), flow did not correlate with performance and emotive dimensions, and emotive dimensions emerged as closely related to flow.

Similar results were reached by (Butkovic et al., 2015), who focused on the relationship between dispositional flow and music practice using an ad hoc questionnaire to assess flow proneness (Swedish Flow Proneness Questionnaire—SFPQ—(Ullén et al., 2012). They analyzed the link between both general flow and “music flow” with music practice. Only the “music flow” experience predicted the music practice (0.41; *p* < 0.001), which in turn predicted the expertise in music (Lehmann and Ericsson, 1997; Ackerman, 2014; Hambrick et al., 2014). Genetic factors mostly explained music flow (40%). Therefore, this study evidenced that flow was an experience less dependent on the environment in which it took place, but it was closely linked to individual proneness (de Manzano et al., 2010).

In particular, Fritz and Avsec (2007) deepened the results reached by Marin and Bhattacharya (2013), exploring the relationship between dispositional flow, positive and negative emotions (using PANAS, Watson et al., 1988) and subjective well-being (using the Satisfaction with Life Scale, Diener et al., 1985). Music performances, concerts and simply playing an instrument or singing are the most flow-conductive activities (22% of flow is experienced in these activities). Also in this study, dimensions of “loss of self-consciousness” and “time transformation” seemed not be related to well-being in a musical context. On the other hand, it seemed that paying less attention to the task, having a clear idea of what happened and feeling a balance between challenges and skills are the keys to experiencing more positive emotions.

Life satisfaction was weakly related to flow, while having clear goals could support a sense of satisfaction in life.

It is possible to conclude by claiming that, according to guidelines provided by (Jackson and Marsh, 1996; Jackson and Eklund, 2002; Jackson et al., 2008, 2010; Martin and Jackson, 2008), flow in musical performance contexts was most investigated in dispositional and emotive terms, and the most investigated target was pianists.

## Conclusion: The Relationship between Flow and Music

The main objective of this review was to explore “state” and “trait” flow in the context of music. To distinguish between flow as a “trait” and as a “state,” we considered guidelines provided by literature concerning flow assessment (Jackson and Marsh, 1996; Jackson and Eklund, 2004; Jackson et al., 2008, 2010; Martin and Jackson, 2008). Further, we also included a study suggested by experts (Butkovic et al., 2015).

Following these premises, a total of 10 studies were included and fully reviewed. To be consistent with the main structure of the review, and in order to give a critical overview of the selected studies, we chose to discuss first studies regarding the domain of musical composition, the musical domain of listening, and finally, regarding musical performance.

Considering the first domain which we analyzed (i.e., musical composition), it seemed that one of the most fascinating issues, which emerged first in this review, was the embodied nature of both music and flow. This issue was explicitly addressed by Nijs et al. (2012) who investigated the relationship occurring among these two phenomena (i.e., flow and music), presence and new technologies. Musicians had the opportunity to use their own bodies to interact with a machine that helped them create their personal music. Nijs et al. (2012) underlined the role of the action-perception coupling principle, and therefore the role of the body, at the base of the relationship between flow and presence.

To understand this perspective, an interesting framework is the enactive approach to cognition by Varela et al. (1991), who stated that our experience is co-built thanks to the reciprocal interactions that occur between mind, body and environment. In these complex reciprocal interactions, our body can act in three different modalities that are closely related. One of these modalities is the “sensorimotor-coupling,” which allows us to have access to the world (perception) supporting an attunement of our body with the environment that facilitates an embodied interaction (Nijs et al., 2012).

Since it is accepted by some scholars that music is an embodied phenomenon (Leman, 2008), studies on the embodied nature of flow are still in the early stages. Nevertheless, in our opinion, sensorimotor-coupling is not only a mechanism that underlines the relationship between flow and presence (Nijs et al., 2012) but it is also the core of flow itself and of its relationship with music. Overcoming the dichotomy between cognition and emotions, it is possible to posit that there are interactions among these poles and environment that bring forth the relationship between flow and music. Therefore, to use a metaphor, the flow-music relationship is mirrored in the famous opera “Psyche Revived by Cupid’s Kiss” by Canova^1^. They don’t need to be together to start existing. Nevertheless, if they met, their reciprocal interactions bring forth a co-built shape of flow and music, allowing them to change their roles and to become at once “amore” and “psyche.” In other words, they definitively give life to an embodied autonomous system in which inner and outer co-exist and are able to interchange their own roles.

However, the issue of the embodied nature of music-flow system needs to be more deepened by future researches in this field which should consider also the peculiarities of the specific musical settings in which this relationship takes place.

For example, Baker and MacDonald clearly evidenced some features which characterized musical composition field. First, in their study it emerged that flow seemed not to be a matter of age. Indeed, no significant differences emerged in experiencing flow (as a state and as a trait) between students and retirees during tasks of musical composition. Therefore, it is necessary for future researches to investigate if this feature characterizes also the other two domains which we considered (i.e., listening and musical performance).

Besides this, flow proneness was able to predict the sensation of having done something meaningful and was more closely related to composition rather than performance (Jackson and Eklund, 2004; Baker and MacDonald, 2013).

A possible explanation of these findings could be found in the work of Baumann (2012). Since autotelic personalities are characterized by both “receptive (e.g., openness) and acetive qualities (e.g., engagement and persistence)” (Baumann, 2012, p. 3), composition allowed “autotelic people” to express both their qualities of “acetors” (i.e., they composed a new excerpt of music) and “receptors” (i.e., diligence and engagement in the task), leading to a feeling of completeness. Indeed, they felt more satisfaction with their product and a greater sense of self-fulfillment that could probably not be reached in a performance context in which musicians and singers (experts or amateurs) usually played something they knew well and that was rarely self-selected.

Together all these observations showed the relevance of deepening the complex interplay between state and trait flow components in music.

With regard to this, a valuable contribution could be found in the field of music listening, which was the second domain of our analysis.

Although it might seem that measuring flow experience in listening to music did not make much sense because flow has been often investigated in “achievement” activities instead of “non-achievement” ones (Schiepe-Tiska and Engeser, 2012), there is clear evidence of the fact that this experience occurred also during simply listening to music (Csikszentmihalyi et al., 1977; Csikszentmihalyi and LeFevre, 1989). Surprisingly, the relationship between music listening and flow was investigated most regarding sportive performance (Karageorghis et al., 2000; Karageorghis and Priest, 2012; Laukka and Quick, 2013) than concerning a more general perspective (Lamont, 2011; Diaz, 2014). Regarding the musical domain, for example, Marin and Bhattacharya’s study referred to the dimension of “music listening” even though this research was not included in that category due to the fact that it focused mostly on musical performance features. Interestingly, it emerged that participants experienced more flow during music listening than during piano performance, maybe because an examination setting was less flow-conductive (Wrigley and Emmerson, 2013).

From these few studies, the existence of a relationship between music listening, flow and performance emerged clearly, even though it appeared all but linear.

Therefore, we chose to integrate findings regarding the domain of music listening and music performance, in order to better clarify the nature of this complex relationship.

First, selected literature regarding music listening suggested that this relationship takes a well-defined shape if the music is self-selected (Pates et al., 2003). As self-selected music was more able to help people to improve their performance, a dispositional explanation seemed best. People’s “own music,” according to personal experience and inclination, could better support performance, maybe because it perfectly met participants’ needs in that specific moment. Indeed, several studies pointed out that self-chosen music had a great impact on emotional states (Mitchell et al., 2007; Sloboda, 2010).

Further, studies regarding the third domain of musical performance suggested that personal emotive intelligence emerged as influencing the strength of the flow experiences we live and as affecting our performance (Marin and Bhattacharya, 2013). Fritz and Avsec (2007) analyzed the relationship between emotions and flow proneness. They evidenced that a person (i) who is prone to have clear in mind what he/she was doing and (ii) who usually felt competent regarding the task could more likely experience positive emotions. On the other hand, paying more attention to a specific task seemed to hinder the emergence of positive emotions.

The predominance of emotive instead of cognitive components of flow seemed to be the key for our personal well-being but not for the reaching of an optimal performance (Bloom and Skutnick-Henley, 2005), in which it was the balance between these two dimensions that played a fundamental role (de Manzano et al., 2010).

Deepening the analysis of the relationship between flow and performance in musical contexts, Butkovic et al. (2015) found that it was “music flow” (i.e., a specific measure of individual flow proneness in music) and not a general predisposition to flow that was the factor able to predict music practice, and therefore, expertise. Besides this, music flow was influenced mainly by a genetic component instead of factors related to a specific situation.

Therefore, while listening to music was found to be closely related to flow (Pates et al., 2003), the link between performance and flow was less clear and needs to be further investigated, and dispositional components of this optimal experience seemed the key to better address this issue (Martin and Jackson, 2008; Sinnamon et al., 2012; Marin and Bhattacharya, 2013; Wrigley and Emmerson, 2013).

Starting from all the above-mentioned findings, it seemed that promising directions for future researchers could be:

• A deeper examination of the role of self-selected music.
• A deeper analysis of the ability not to let emotive components overcome cognitive mechanisms during task execution (e.g., “concentration,” “sense of perceived control” and “clear goals”), which refers to the general competence of managing them adequately (Wilson and Roland, 2002; Fullagar et al., 2013), because they seemed the keystones of the complex architecture that supports the link between flow and performance in the musical domain.
• New ways to address dispositional aspects of flow in musical contexts, maybe considering also the impact of genetic factors determining flow.

To address the second above-mentioned issue concretely (i.e., adequate managing of emotive components of flow), we referred to literature concerning anxiety and musical performance (Wilson and Roland, 2002; Ryan, 2004; Kirchner, 2005, 2011; Fullagar et al., 2013). As Lamont (2012) clearly suggested, focusing on negative emotive components occurring during music performance, such as anxiety, biofeedback training and the accurate selection of the excerpts of music to be played, could be appropriate techniques to successfully face performance anxiety.

In our view, it could be useful to help musicians (or singers, of course) to use biofeedback training in order to adequately manage positive emotions that could emerge during performance. Indeed, positive affects could divert the executor’s attention from the specific task, hindering the emergence of cognitive dimensions of flow, such as concentration, clear goals and sense of control on task. In this case, the appropriate balance between emotive and cognitive dimensions of flow would emerge with difficulty, hindering the reaching of high performance levels.

Since affects seemed crucial in order to investigate both dispositional and state flow in musical contexts, a further integration in this direction could be considering also music-induced emotions and not only emotive components of flow, giving their relevance in both phenomena (i.e., music and flow). For example, Juslin (2013, p. 240) developed a model regarding music-evoked emotions in which the psychophysiological dimension was also considered. In a broad perspective, Goffin (2014) showed that music evokes bodily feelings that can be clustered into specific moods and can influence the esthetic appreciation of music.

Therefore, it would be very useful to consider music-induced emotions in relation with emotive components of flow experience. Among the selected studies, only Pates et al. (2003) attempted to focus also on music in these terms, while other researches concentrated only on emotional components of flow. This approach could be useful in building an embodied vision of flow in music, allowing all the main components characterizing this process to be considered together (Goffin, 2014; Harrison and Loui, 2014).

To address the third above-mentioned issue, because it seemed that the most fruitful approach to be pursued in the musical domain is the dispositional one (Bloom and Skutnick-Henley, 2005; Kirchner, 2005, 2011; Sinnamon et al., 2012; Baker and MacDonald, 2013; Butkovic et al., 2015) we suggest that the analysis of flow relative to a specific situation should always be followed by a check of each participant’s flow proneness.

Additionally, in the musical domain, it could be more opportune to overcome the simple investigation of the causes fostering flow in a specific situation, maybe by analyzing factors that could influence our proneness to flow, such as genetic factors or simply self-trust or self-confidence as personality traits (Bloom and Skutnick-Henley, 2005).

The dispositional approach should be the first step in each study because it could help researchers focus on stable and cross-situational factors underpinning flow. The second stage could utilize a situational perspective after we had focused on a specific situation and had defined it accurately.

Following this process, researchers should keep in mind that dimensions that characterize flow (i.e., the nine flow dimensions) are only flow components and not factors able to induce flow in a specific situation (Schiepe-Tiska and Engeser, 2012). Therefore, the gaze should be broader, overcoming flow components and searching for situational or dispositional factors external to flow dimensions (Bakker, 2005; Schiepe-Tiska and Engeser, 2012).

For example, the original form of the Experience Sampling Method (ESM), introduced by Csikszentmihalyi et al. (1977), Csikszentmihalyi and Larson (1987) and indicated as a dispositional measure of flow by (Baumann, 2012), could be enriched by introducing psychophysiological measures of flow, as Gaggioli et al. (2013a) successfully did.

The same Dispositional Flow State Scale should be revised because it assesses mainly the frequency with which an individual experienced each of the dimensions of flow. It might be possible to enlarge the viewpoint, overcoming the “frequency” perspective as an indicator of flow proneness and, generally, adopting a longitudinal perspective toward flow in musical contexts, as Custodero successfully did (Custodero, 1998, 2002, 2005, 2012).

Further, regarding flow assessment specifically, it emerged that dimensions of “time transformation” and “loss of self-consciousness” seemed the least relevant in the musical domain from both a state and a dispositional perspective (Fritz and Avsec, 2007; Sinnamon et al., 2012; Wrigley and Emmerson, 2013). It would be useful to understand why these Flow dimensions seemed so much less relevant in a domain to which they are usually naïvely associated (Schäfer et al., 2013). It is possible that current instruments are not adequate to properly assess these dimensions. Also in this case, an integration of a self-reported questionnaire with other psychophysiological measures could be a practicable direction (Gaggioli et al., 2013a).

Moreover, another future challenge for research in this field could be a group perspective of analysis toward flow in music. Indeed, in all the selected studies the relationship between music and flow has been investigated mainly at the individual level (Pates et al., 2003; Nijs et al., 2012), even though a person could often live a musical experience along with others (e.g., consider choirs or orchestras, musical bands or simply listening to music with friends).

Therefore, it could be interesting to analyze flow in musical settings from a group perspective, as was successfully done by (Aubé et al., 2014), although in a different context (i.e., teamwork). On the other hand, this focus on group level seemed to be a trend mainly followed by researchers interested in creativity. This could be due to the fact that recently creativity has been seen as a group phenomenon, and therefore several models on creativity were born starting from these premises (Sawyer, 2006, 2008; Gaggioli et al., 2013b).

This is the case of the Networked Flow model recently developed by (Gaggioli et al., 2013b), which can be considered as a new approach to investigate the relationship between music and flow in group terms and starting from validated instruments (i.e., FSS is usually employed to assess flow after a specific performance). In particular, according to this model, at a group level it is crucial to consider another type of optimal experience labeled “group flow” (Sawyer, 2003, 2008), which is supposedly able to support excellent performances, as Hart and Di Blasi (2015) proved using semi-structured interviews.

Therefore, a conclusive future challenge could be considering together two main aspects of exploring flow in musical contexts. The first are emotional components of both music and flow, while the second is a group level of analysis that is nearly unexplored in a musical context. The Networked Flow model seemed to offer the possibility of addressing all the above-mentioned issues (i.e., emotive components and group level of analysis), (i) starting from concepts of Social Presence (Biocca and Harms, 2003; Riva, 2008; Riva et al., 2011), which also encompasses an embodied perspective on emotive dimension (i.e., emotive contagion), and (ii) referring to the above-mentioned group flow that broadens the perspective to a group level.

## Limitations

The limitations of the present review concerned mainly the small number of studies included, which were not intended to be representative of the whole field of “flow in music.” Nevertheless, all the studies fully met the inclusion criteria, which were accurately selected in order to focus on a small proportion of studies concerning dispositional and state flow in the musical domain.

Moreover, another clear limitation is due to the retrospective nature of the instruments used in these studies.

### Conflict of Interest Statement

The authors declare that the research was conducted in the absence of any commercial or financial relationships that could be construed as a potential conflict of interest.

## Footnotes

1 Canova, A. (1757–1822). *Psyche Revived by Cupid’s Kiss* Paris: Louvre.
